# Fatty Acid‐Binding Protein 5 Potentially Regulates the Proliferation and Metastasis of Uveal Melanoma Cells Through the PI3K/AKT/mTOR Signaling Pathway

**DOI:** 10.1002/cns.71112

**Published:** 2026-09-04

**Authors:** Yue Du, Xue Jiang, Yanyan Zhang, Jianing Ying, Quanyong Yi

**Affiliations:** ^1^ Ningbo Key Laboratory of Medical Research on Blinding Eye Diseases, Ningbo Eye Institute, Ningbo Eye Hospital Wenzhou Medical University Ningbo China

**Keywords:** *FABP5*, immune infiltration, machine learning, PI3K/AKT/mTOR, tumor model, uveal melanoma

## Abstract

**Background:**

Uveal melanoma (UVM) is an aggressive intraocular tumor with limited effective treatments for metastatic disease. This study explored the pathways through which fatty acid‐binding protein 5 (*FABP5*) promoted UVM progression.

**Methods:**

Single‐cell RNA sequencing datasets were preprocessed by the Seurat package, and prognostic genes were screened using the survival package. A prognostic model was constructed via LASSO Cox regression, followed by Kaplan–Meier (KM) survival analysis. To characterize the tumor immune microenvironment, we evaluated immune cell infiltration using ssGSEA, ESTIMATE, and CIBERSORT algorithms, while GSEA was applied to identify enriched pathways. Additionally, immunotherapy response was predicted by TIDE, and tumor mutation burden (TMB) was calculated using Maftools. For in vivo validation, a nude mouse xenograft model was established, and Western blotting was performed on tumor tissues harvested from the xenograft assay.

**Results:**

*SPON2*, *MATK*, and *FABP5* were identified as hub genes in UVM and used to construct a prognostic model with a high AUC. Immune infiltration analysis revealed that high‐risk patients exhibited features of immune evasion. Enrichment analysis further revealed that cytotoxic T‐cell‐related genes (*MATK* and *FABP5*) were enriched in the PI3K/AKT/mTOR pathway. *FABP5* was overexpressed in UVM cells, whereas *FABP5* silencing suppressed cell viability, proliferation, migration, and invasion, reduced MMP2 and MMP9 levels, promoted apoptosis, downregulated PI3K/AKT/mTOR pathway‐related molecules, and upregulated apoptosis‐related proteins. Notably, these effects could be reversed by the PI3K agonist 740Y‐P.

**Conclusion:**

*FABP5* promoted UVM cell proliferation and migration through the PI3K/AKT/mTOR pathway. Though the immune‐infiltration analysis was hypothesis‐generating, it may help identify patients likely to benefit from immunotherapy.

## Graphical abstract

Single‐cell and bulk sequencing reveal *SPON2*, *MATK*, and *FABP5* as prognostic indicators in uveal melanoma. We demonstrated that *FABP5* promotes malignancy via the PI3K/AKT/mTOR pathway, as genetic knockdown impairs tumor growth and is rescued by 740Y‐P pharmacological pathway activation. These findings highlight *FABP5* maybe as a critical driver of immune evasion and a potential biomarker for refining patient selection in future immunotherapeutic trials.

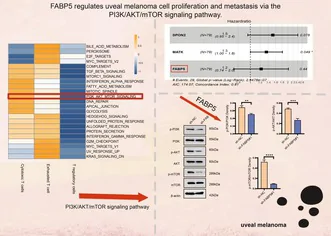

**Abbreviations**
AKT: protein kinase B
ANOVA: analysis of variance
AUC: area under the curve
BsAb: bispecific antibody
CCK‐8: cell counting kit‐8
DMEM: Dulbecco's Modified Eagle's Medium
DMEM/F12: Dulbecco's Modified Eagle's Medium/Nutrient Mixture F‐12
ECM: extracellular matrix
FABP5: fatty acid‐binding protein 5
FBS: fetal bovine serum
GEO: Gene Expression Omnibus
ICI: immune checkpoint inhibitors
KM: Kaplan–Meier
LASSO: Least Absolute Shrinkage and Selection Operator
MATK: mast cell tyrosine kinase
MMP: matrix metalloproteinase
MSigDB: Molecular Signatures Database
mTOR: mammalian target of rapamycin
NF‐κB: nuclear factor‐κB
OD: optical density
OS: overall survival
P/S: penicillin–streptomycin solution
PBS: Phosphate Buffered Saline
PCA: principal component analysis
PD‐1/PD‐L1: Programmed Cell Death Protein 1/Programmed Cell Death Ligand 1
PFI: Progression‐Free Interval
PI3K: phosphatidylinositol 3‐kinase
PVDF: Polyvinylidene Fluoride
qRT‐PCR: Quantitative Reverse Transcription PCR
RNA‐seq: RNA sequencing
ROC: receiver operating characteristic
RPMI‐1640: Roswell Park Memorial Institute 1640 Medium
scRNA‐seq: single‐cell RNA sequencing
SD: standard deviation
SDS‐PAGE: Sodium Dodecyl Sulfate‐Polyacrylamide Gel Electrophoresis
SPON2: spondin 2
ssGSEA: single‐sample gene set enrichment analysis
STR: short tandem repeat
TAM: tumor‐associated macrophage
TCGA: The Cancer Genome Atlas
TIDE: Tumor Immune Dysfunction and Exclusion
TMB: tumor mutational burden
TME: tumor microenvironment
TPM: Transcripts Per Million
UMAP: Uniform Manifold Approximation and Projection
UVM: uveal melanoma
VEGF: vascular endothelial growth factor
WB: Western blotting

## 1 Introduction

Uveal melanoma (UVM) is the most common primary malignancy of the eye. It arises mainly from the choroid (90%), followed by melanocytes of the ciliary body (6%) and the iris (4%) [1]. Compared with cutaneous melanoma, UVM exhibits distinct molecular drivers [2] and is characterized by high metastatic potential and poor prognosis [3]. Although the incidence of UVM remains relatively low and the majority of primary lesions can be treated with radiotherapy, enucleation, or other interventions, more than 40% of patients eventually develop distant organ metastases [4], with a pronounced tendency for hepatic dissemination [5]. Emerging evidence indicates that UVM metastasis is intricately linked to tumor immune microenvironment regulation and gene expression reprogramming [6, 7]. However, due to a lack of standard treatment regimens for metastatic UVM [8], elucidating the mechanisms of metastasis and identifying potential therapeutic targets are critical for developing clinical intervention strategies.

Previous findings suggest that identifying T‐cell‐related genes may contribute to the therapeutic development for metastatic UVM [9, 10]. Notably, several T‐cell‐associated genes have been proven to prolong overall survival in patients with metastatic UVM [11]. Nevertheless, their therapeutic benefits are limited to partial patient populations, highlighting the urgent need to identify T‐cell‐related biomarkers to optimize patient stratification. Meanwhile, the PI3K/AKT/mTOR pathway, a crucial and highly complex signaling cascade that involves over 150 regulatory proteins [12], is aberrantly activated in many cancers. As a central effector molecule in PI3K signaling [13], AKT phosphorylation triggers mTORC1 activation and suppresses autophagy, thereby promoting cell growth and proliferation [14]. Therefore, exploring how the PI3K/AKT/mTOR signaling cascade interacts with T‐cell‐related genes may help clarify the mechanisms of UVM proliferation and guide targeted treatments.

Among the T‐cell‐related candidates, fatty acid‐binding protein 5 (*FABP5*) is of particular interest. As a small intracellular lipid chaperone, *FABP5* transports long‐chain fatty acids and related lipids to specific cellular sites [15]. Critically, it delivers fatty acids and retinoic acid to peroxisome proliferator‐activated receptor β/δ (PPARβ/δ), shifting retinoic acid signaling away from growth suppression toward the activation of genes that favor proliferation and survival [16]. *FABP5* also engages PPARγ to reprogram lipid metabolism, meeting the elevated energy and membrane demands of tumor cells [17]. Consequently, *FABP5* promotes epithelial‐mesenchymal transition (EMT) via upregulation of SNAI1 and β‐catenin signaling [18], enhances PI3K/AKT/mTOR signaling [19, 20, 21], and remodels the tumor immune microenvironment, as *FABP5*‐PPARγ signaling in lipid‐loaded macrophages weakens T‐cell antitumor immunity [22]. Beyond these pathways, *FABP5* drives tumor progression through upregulating vascular endothelial growth factor (VEGF) to facilitate angiogenesis, increasing matrix metalloproteinases (MMPs) to degrade the extracellular matrix, inducing de novo fatty acid synthesis, and activating the nuclear factor‐κB (NF‐κB) inflammatory signaling pathway [23]. Elevated *FABP5* expression is related to a poor prognosis in liver, prostate, and digestive‐tract cancers [18, 24, 25], and is similarly recognized as a marker of adverse outcomes in UVM [26]. Collectively, these findings position *FABP5* as a candidate for further functional study in UVM.

This study was conducted following the guideline of the TITAN 2025 paper. Based on multi‐omics data, we screened potential targets and performed enrichment analysis. Given that *FABP5* and related T cell subsets are significantly enriched in the PI3K/AKT/mTOR pathway, a cascade closely associated with UVM pathogenesis [27], we examined whether *FABP5* influenced the UVM development by modulating the PI3K/AKT/mTOR pathway at both the mRNA and protein levels. Functional rescue experiments using the PI3K agonist 740Y‐P were also performed to validate these findings.

## 2 Material and Methods

This work was reported in accordance with the ARRIVE Guidelines (Animal Research: Reporting of In Vivo Experiments) [28].

### 2.1 Bioinformatics Analyses

#### 2.1.1 Data Sources

The single‐cell RNA sequencing (scRNA‐seq) dataset (GSE139829) containing eight primary tumor samples and three metastatic samples was obtained from the Gene Expression Omnibus (GEO) database. The RNA‐seq expression profiles and clinical follow‐up data of UVM patients were retrieved from The Cancer Genome Atlas (TCGA). The TCGA data were converted to Transcripts Per Million (TPM) format, log_2_‐transformed, and samples with complete progression‐free interval (PFI) information were retained, yielding 79 UVM samples as the training set. Additionally, the GSE22138 dataset containing 63 UVM samples was also obtained from the GEO as the validation set. Probes were mapped to GeneSymbol, and for genes with multiple probes, the mean expression value was calculated.

#### 2.1.2 Processing of the scRNA‐seq Data

The scRNA‐seq data for each sample in GSE139829 were imported using the Read10X function in the Seurat package [29]. Cells with 200–1500 detected genes and mitochondrial genes < 10% were retained, and the SCTransform function was used for normalization [29]. Principal component analysis (PCA) was performed for dimensionality reduction, and batch effects were corrected using the Harmony package [30]. Uniform Manifold Approximation and Projection (UMAP) was then applied for further dimensionality reduction, and cells were clustered utilizing FindNeighbors and FindClusters [29] (dims = 1:20 and resolution = 0.1). Cellular subpopulations were annotated with marker genes from the CellMarker2.0 database [31]. In TCGA cohort, T cells were re‐clustered into three subsets, and single‐sample gene set enrichment analysis (ssGSEA) was used to score each subset based on the high‐expression markers identified from the scRNA‐seq data. Finally, the AUCell algorithm was utilized to compare pathway enrichments among the three T cell subsets [32, 33].

#### 2.1.3 Establishment of a Prognostic Model

Univariate Cox regression analysis was performed using the survival package to identify prognostic genes (*p* < 0.05) in UVM [34]. The resulting candidate gene set was further refined by LASSO Cox regression with the glmnet package [35]. Stepwise regression was applied to select the final combination of key genes to develop a RiskScore formula: RiskScore=Σβi×Expi. Based on the median RiskScore, patients were divided into high‐risk and low‐risk groups. The model's predictive performance was assessed using Kaplan–Meier (KM) survival analysis and receiver operating characteristic (ROC) curve generated with the timeROC package [25], with external validation on an independent dataset.

#### 2.1.4 Analysis of Immune Infiltration and Immunotherapeutic Responses

Immune infiltration was evaluated by the ssGSEA algorithm with immune cell gene signatures from published studies [32, 36]. The ESTIMATE and CIBERSORT algorithms were applied to quantify the immune and stromal components in the tumor microenvironment (TME) [37]. Response to immune checkpoint inhibitors (ICIs) was predicted by the Tumor Immune Dysfunction and Exclusion (TIDE) algorithm (http://tide.dfci.harvard.edu/). Tumor mutational burden (TMB) was calculated using the Maftools package based on somatic mutation profiles derived from the TCGA‐UVM cohort [38].

#### 2.1.5 GSEA

Pathway enrichment analysis was performed using the GSEA algorithm against the HALLMARK gene sets from the Molecular Signatures Database (MSigDB, https://www.gsea‐msigdb.org/gsea/msigdb/) [39]. Hallmark pathway scores were calculated via ssGSEA implemented in the GSVA package [32].

### 2.2 Cell Culture and Transfection

Human retinal pigment epithelial cell line ARPE‐19 (control group) (CL‐0026, Procell, Wuhan, China) was incubated in Dulbecco's Modified Eagle's Medium/Nutrient Mixture F‐12 (DMEM/F12) medium (PM150312, Procell) [40]. Human UVM cell lines Mel270 (primary UVM) (CL‐0994, Procell) and OMM2.3 (metastatic UVM) (CC1809, Cellcook, Guangzhou, China) (https://www.cellcook.com/) were cultured in Roswell Park Memorial Institute 1640 Medium (RPMI‐1640) (PM150110, Procell). MUM‐2B (metastatic UVM) (BFN60700331, Bluefcell, Shanghai, China) (http://www.bluefcell.com/) [41] was cultured in high‐glucose DMEM (PM150210, Procell). All media contained 10% fetal bovine serum (FBS, 164210, Procell) and 1% penicillin–streptomycin solution (P/S, PB180120, Procell). Cell lines were validated through short tandem repeat (STR) profiling, confirmed to be free of mycoplasma, and cultured at 37°C in a humidified environment with 5% CO_2_.

For rescue assays, the PI3K activator 740Y‐P (HY‐P0175, MedChemExpress, Monmouth Junction, NJ) was dissolved in sterile distilled water to prepare a 1 mg/mL stock solution (filtered through a 0.22 μm membrane) and then diluted to a final working concentration of 10 μg/mL (≈3.056 μM). Cells were seeded at 2 × 10^5^ cells per well in 6‐well plates 24 h prior to drug administration, and 740Y‐P was added when cells reached 60%–70% confluence. Cells were pretreated with 740Y‐P for 1 h before sh‐*FABP5* transfection, and the drug‐supplemented medium was replaced every 24 h to maintain stable pathway activation throughout the 48‐h treatment period. Control groups received an equivalent volume of sterile distilled water [42, 43].

For transfection, Lipofectamine 2000 reagent (11668027, Invitrogen, Carlsbad, CA) was used in accordance with the manufacturer's instructions to deliver target sequences of sh‐*FABP5*#1 (5′‐GTATCATCACTTGTGATGGTA‐3′) and sh‐*FABP5*#2 (5′‐GCAACTTTACAGATGGTGCAT‐3′) (Sigma‐Aldrich, St. Louis, MO) into MUM‐2B and OMM2.3 cells, with a non‐targeting short hairpin RNA (sh‐NC) as the negative control. After transfection, the cells were incubated at 37°C for 48 h.

### 2.3 Xenograft Mouse Model

Five‐ to six‐week‐old male BALB/c nude mice (SPF grade, weighing 18–22 g) were purchased from Beijing Vital River Laboratory Animal Technology Co. Ltd. and housed in a specific‐pathogen‐free facility under controlled conditions (temperature 22°C ± 2°C, humidity 50% ± 10%, 12‐h light/dark cycle) with free access to sterilized food and water.

MUM‐2B cells stably expressing sh‐NC or sh‐*FABP5*#1 were established by lentiviral infection followed by puromycin selection (1 μg/mL, 7 days) to obtain polyclonal stable pools. Before injection, cells were trypsinized and washed once with sterile PBS (C0221A, Beyotime, Shanghai, China). Mice were randomly assigned into two groups (*n* = 6 per group, balanced for body weight) and received a subcutaneous injection of 1 × 10^6^ cells in 50 μL PBS into the left posterior abdomen [44]. Tumor length (L, the longest diameter) and width (W, the perpendicular diameter) were measured every 3 days using Vernier calipers, and tumor volume (V) was calculated as V (mm^3^) = (L × W^2^) / 2. Body weight was monitored simultaneously. Mice were euthanized by cervical dislocation under isoflurane anesthesia on day 24 post‐injection, or earlier if any tumor exceeded 1500 mm^3^, became ulcerated, or if the mouse reached humane endpoints (≥ 20% body weight loss, severe cachexia, or impaired mobility). Excised xenografts were photographed, weighed, and processed for subsequent analysis.

### 2.4 Quantitative Reverse Transcription PCR (qRT‐PCR)

qRT‐PCR was performed to determine *FABP5* expression in each cell line, and the knockdown effect of *FABP5* was confirmed. Total RNA was initially extracted using TRIzol reagent (15596026CN, Invitrogen), and its concentration and purity were measured using a microplate reader (ThermoFisher Scientific, Waltham, MA). After removing genomic DNA contamination, we used the BeyoRT II cDNA Synthesis Kit (D7170M, Beyotime) to synthesize cDNA. Additionally, qRT‐PCR was carried out with BeyoFast SYBR Green qPCR Mix (D7265, Beyotime) on the ABI GeneAmp 5700 Sequence Detection System (Applied Biosystems, Billings, MT) [45]. The primer sequences were as follows: *FABP5* (NM_001444) forward GGTGCATTGGTTCAGCATCAGG and reverse TCATAGATCCGAGTACAGGTGAC; *GAPDH* (NM_002046) forward GTCTCCTCTGACTTCAACAGCG and reverse ACCACCCTGTTGCTGTAGCCAA.

### 2.5 Western Blotting (WB)

WB was performed to detect PI3K/AKT/mTOR pathway components (p‐PI3K/PI3K, p‐AKT/AKT, p‐mTOR/mTOR) and cell apoptosis‐related proteins (Bax, cleaved caspase‐3, cleaved caspase‐9, Bcl‐2). These proteins were examined in *FABP5*‐silenced UVM cells treated with or without 740Y‐P, and in xenograft tumor tissues from mice inoculated with *FABP5*‐knockdown cells. Total cellular proteins were extracted using RIPA lysis buffer (89901, ThermoFisher Scientific), and protein concentration was detected with a BCA protein assay kit (GK10009, GlpBio, Montclair, CA) to normalize loading. Equal amounts of protein samples were separated by SDS‐PAGE and transferred to PVDF membranes (FFP32, Beyotime). The membranes were then blocked with 5% non‐fat milk for 2 h to prevent non‐specific binding, and then incubated with primary antibodies (Table S1) overnight at 4°C. After washing, the membranes were incubated with goat anti‐rabbit IgG H&L (HRP) (1:2500, ab205718, Abcam, Cambridge, UK) for 1.5 h at room temperature. Protein bands were visualized using BeyoECL Plus (P0018S, Beyotime), and band intensities were quantified by ImageJ software (Version 1.52).

### 2.6 Cell Counting Kit‐8 (CCK‐8) Assay

CCK‐8 test was used to assess the viability of *FABP5‐silenced* UVM cells with or without 740Y‐P treatment. Specifically, following the indicated treatments, UVM cells (1 × 10^5^ cells/well) were seeded into plates and incubated with 10 μL of CCK‐8 reagent (C0037, Beyotime) for 2 h at 37°C. The absorbance at 450 nm (optical density, OD) was measured using a NanoDrop spectrophotometer (ThermoFisher Scientific).

### 2.7 ELISA

Additionally, ELISA kits were used to determine the levels of *MMP2* (KHC3081, Invitrogen) and *MMP9* (BMS2016‐2, Invitrogen) in UVM cell culture supernatants [46].

### 2.8 Cell Cloning

For the colony formation assay, *FABP5‐*transfected UVM cells treated with/out 740Y‐P were seeded into 6‐well plates at a density of 1 × 10^3^ cells/well in 2 mL of medium. The plates were gently swirled to ensure uniform cell dispersion and incubated at 37°C, with the medium refreshed every 2 days. After 14 days of culture, when visible colonies appeared, the medium was aspirated, and the cells were fixed with 4% paraformaldehyde (P0099, Beyotime) for 15 min, stained using 0.1% crystal violet (C0121, Beyotime) for 15 min, washed three times with PBS (C0221A, Beyotime), and photographed [47].

### 2.9 Cell Migration Assay

Wound healing experiment was performed to assess the migration of *FABP5*‐transfected UVM cells (MUM‐2B and OMM2.3) treated with or without 740Y‐P. Briefly, transfected cells were seeded uniformly into 6‐well plates. Upon reaching full confluence, a scratch was created using a 100‐μL pipette tip. Wound regions were imaged using a microscope (Olympus, Tokyo, Japan) at 0 h and 48 h after scratching. Wound closure was quantified using ImageJ software by measuring the wound width change relative to 0 h [48].

### 2.10 Cell Invasion Assay

The invasion of *FABP5‐transfected* UVM cells treated with/out the presence of 740Y‐P was evaluated using a Transwell assay. Briefly, transfected cells were added into the upper chamber of Transwell inserts (pore size: 8 μm, 3422, Corning Inc., Corning, NY) pre‐coated with Matrigel (C0372, Beyotime), while the lower compartment was filled with complete medium. After 48 h of incubation, cells that had invaded through the membrane were fixed by 4% paraformaldehyde (P0099, Beyotime) and stained by 0.1% crystal violet (C0121, Beyotime). The number of invaded cells was counted with an Olympus microscope (Tokyo, Japan).

### 2.11 Cell Apoptosis Assay

Apoptosis of *FABP5‐transfected* UVM cells treated with/out 740Y‐P was evaluated by flow cytometry. Cells were digested with 0.25% trypsin, rinsed in PBS (C0221A, Beyotime), and then stained by the Annexin V‐FITC Apoptosis Detection Kit (C1062S, Beyotime). The percentage of apoptotic cells in each group was determined using a flow cytometer (BD Biosciences, San Jose, CA) [49].

### 2.12 Statistical Analysis

All statistical analyses were performed utilizing R package (version 3.6.0) and GraphPad Prism (version 8.0.2). Differences in continuous variables between two groups were assessed using the Wilcoxon rank‐sum test, and survival differences across patient subgroups were assessed using the log‐rank test. Spearman's correlation analysis was conducted to examine relationships between variables. Unpaired *t*‐tests were used for two‐group comparisons, and one‐way or two‐way ANOVA with Bonferroni's multiple comparisons test was performed for comparisons involving three or more groups. Data from three biological replicates per group are presented as mean ± standard deviation (SD). A *p*‐value < 0.05 was considered statistically significant.

## 3 Results

### 3.1 Single‐Cell Atlas of UVM and a Risk Model for UVM Prognosis

The results demonstrated that the proportion of T cells was higher in metastatic samples than in primary tumor samples (Figure S1A,B). Among these, only cytotoxic T cells were significantly associated with PFI and were therefore identified as an independent prognostic predictor of PFI (*p* < 0.05, Figure S1C). Survival analysis revealed that patients with high cytotoxic T‐cell infiltration had significantly shorter PFI (*p* < 0.05, Figure S1D). Pathway enrichment analysis showed that cytotoxic T cells were enriched in signaling pathways including PI3K/AKT/mTOR (Figure S1E).

Three key genes were identified to construct a prognostic model using the formula: RiskScore=0.415*SPON2+0.297*MATK+0.199*FABP5 (Figure S2A–C). TCGA samples were divided into high‐risk and low‐risk subgroups by the median RiskScore. ROC analysis yielded a high area under the curve (AUC), suggesting robust predictive performance of the model (Figure S2D). Patients with higher RiskScores showed significantly worse PFI (*p* < 0.05, Figure S2E), and all three genes were highly expressed in the group with high RiskScores (*p* < 0.05, Figure S2F). External validation using the GSE22138 cohort further confirmed the model's prognostic value (*p* < 0.05, Figure S2G–I).

### 3.2 Infiltration, Immunotherapy Response, and GSEA

The high‐risk group exhibited higher ImmuneScore and StromalScore, indicating increased stromal components and immune cell infiltration in the TME (*p* < 0.05, Figure S3A). Additionally, the expression of the three key genes showed significant positive correlations with ImmuneScore, StromalScore, and ESTIMATEScore (*p* < 0.05, Figure S3B). The high‐risk group displayed markedly elevated enrichment scores for multiple immune cell subsets as well as higher TIDE scores (Figure S3C–E). Consistent with this, the ICI response rate was markedly lower in the high‐risk group (Figure S3F), along with significantly increased overall TIDE scores (*p* < 0.05, Figure S3G). TMB analysis indicated that the low‐risk group had higher TMB (*p* < 0.05, Figure S3H). Notably, multiple immune checkpoint genes were markedly upregulated in the high‐risk group (Figure S3I). In addition, Hallmark pathway analysis revealed that the expression of *MATK* and *FABP5* was significantly positively correlated with the PI3K/AKT/mTOR signaling pathway activity (Figure S4A,B).

### 3.3 The Expression of *FABP5* in UVM Cells

**FIGURE 1:**
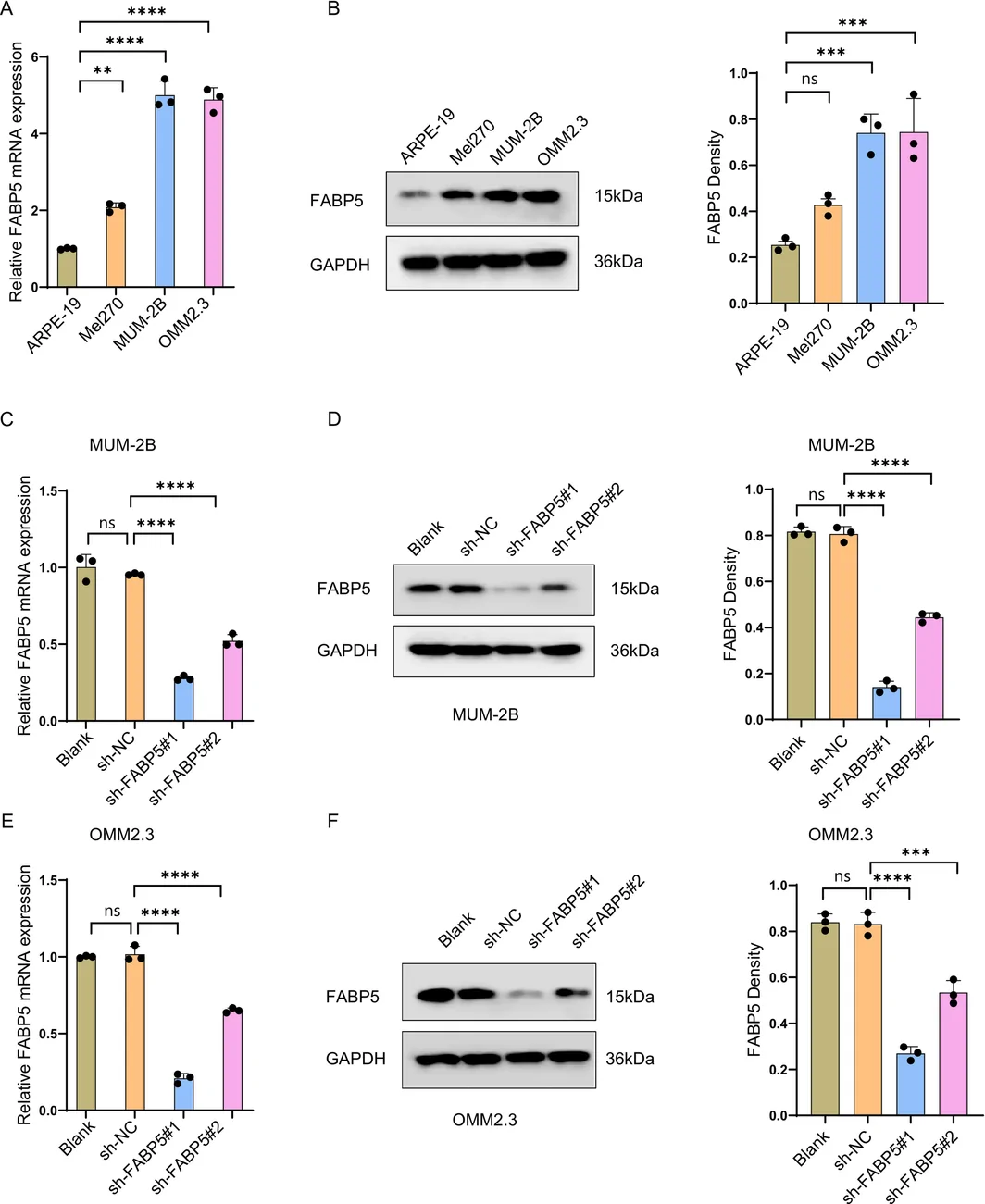
Role and transfection of *FABP5*. (A‐B) Expression of *FABP5* in ARPE‐19, Mel270, MUM‐2B, and OMM2.3 cell lines, as quantified in the assays of (A) qRT‐PCR and (B) WB. (C‐D) Transfection of short hairpin RNA targeting *FABP5* in MUM‐2B cells with (C) qRT‐PCR and (D) WB validation on the silencing efficiency. (E‐F) Transfection of short hairpin RNA targeting *FABP5* in OMM2.3 cells with (E) qRT‐PCR and (F) WB validation on the silencing efficiency. ns means not significant, * means *p* < 0.05, ** means *p* < 0.01, *** means *p* < 0.001, **** means *p* < 0.0001.

qRT‐PCR results revealed significantly increased *FABP5* expression in all UVM cell lines, with metastatic OMM2.3 and MUM‐2B cells exhibiting higher expression than the primary Mel270 cells (*p* < 0.05, Figure 1A). WB analysis confirmed that *FABP5* protein levels were also higher in metastatic OMM2.3 and MUM‐2B cells compared with Mel270 cells (*p* < 0.05, Figure 1B). Both qRT‐PCR and WB confirmed successful *FABP5* knockdown after transfection of sh‐*FABP5* into OMM2.3 and MUM‐2B cells. sh‐*FABP5*#1, which achieved greater knockdown efficiency, was selected for subsequent experiments (*p* < 0.05, Figure 1C–F).

### 3.4 Cellular Changes Following Sh‐ *FABP5* Transfection

**FIGURE 2:**
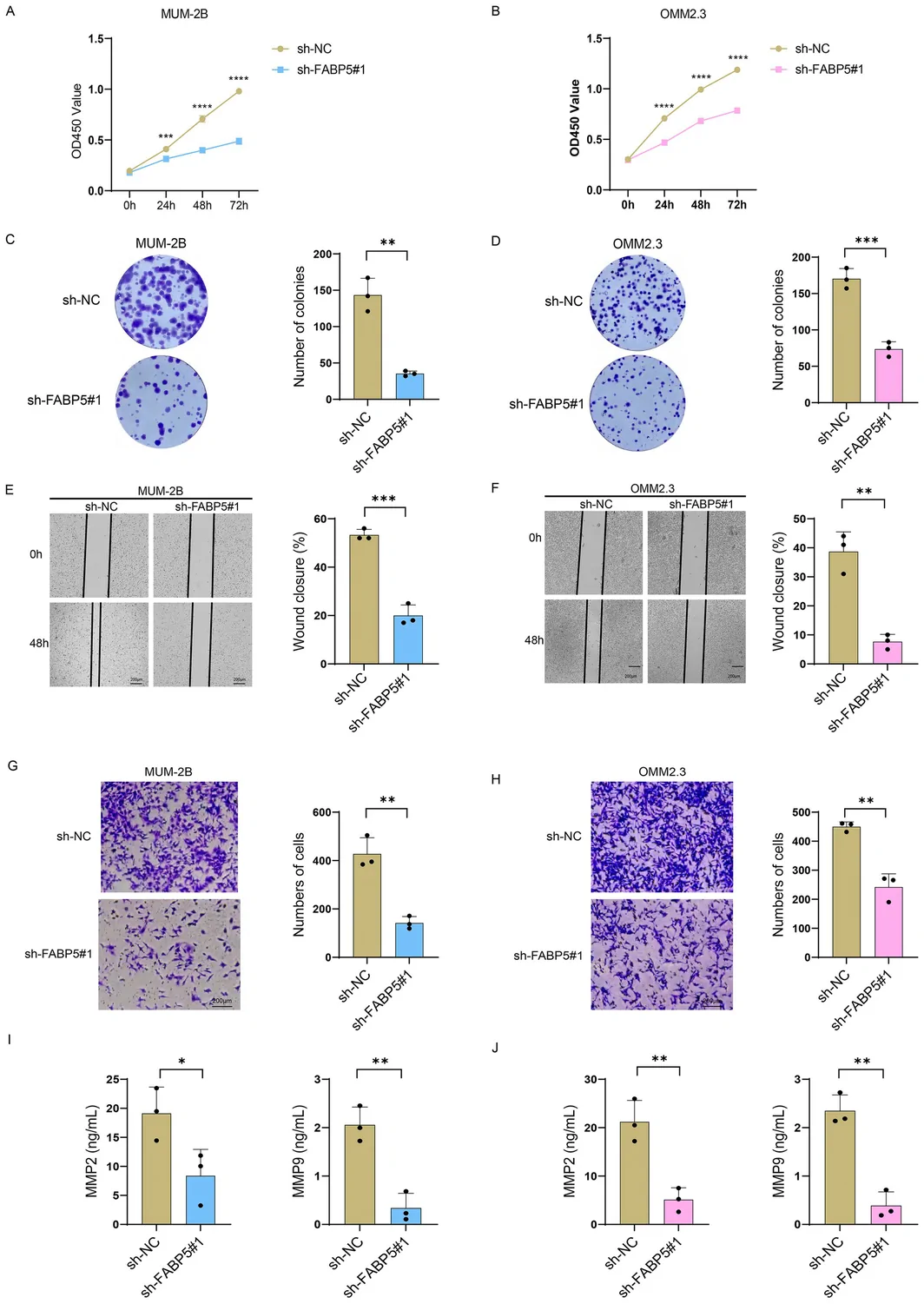
Cellular phenotypes after sh‐*FABP5* transfection. (A‐B) CCK‐8 assay for cell viability after sh‐*FABP5* transfection in (A) MUM‐2B and (B) OMM2.3 cell lines. (C‐D) Colony formation assay for proliferative capacity after sh‐*FABP5* transfection in (C) MUM‐2B and (D) OMM2.3 cell lines. (E‐F) Wound healing assay for migration ability after sh‐*FABP5* transfection in (E) MUM‐2B and (F) OMM2.3 cell lines. (G‐H) Transwell invasion assay for invasive capacity after sh‐*FABP5* transfection in (G) MUM‐2B and (H) OMM2.3 cell lines. (I‐J) Levels of MMP2 and MMP9 after sh‐*FABP5* transfection in (I) MUM‐2B and (J) OMM2.3 cell lines. ns means not significant, * means *p* < 0.05, ** means *p* < 0.01, *** means *p* < 0.001, **** means *p* < 0.0001.

CCK‐8 assay revealed that the viability of sh‐*FABP5*#1‐transfected OMM2.3 and MUM‐2B cells was markedly lower than that of the sh‐NC group (*p* < 0.05, Figure 2A,B). Colony formation assays showed markedly reduced proliferation in the sh‐*FABP5*#1 groups compared with the sh‐NC group (*p* < 0.05, Figure 2C,D). Wound healing assay demonstrated that cell migration was significantly inhibited in the sh‐*FABP5*#1 group (*p* < 0.05, Figure 2E,F). Consistently, Transwell assay confirmed that the invasion of *FABP5*‐silenced UVM cells was also suppressed (*p* < 0.05, Figure 2G,H). Additionally, *MMP2* and *MMP9* level was markedly downregulated in the sh‐*FABP5*#1 group relative to the sh‐NC group (*p* < 0.05, Figure 2I,J).

### 3.5 Association of *FABP5* With the PI3K/AKT/mTOR Pathway and Cell Apoptosis

**FIGURE 3:**
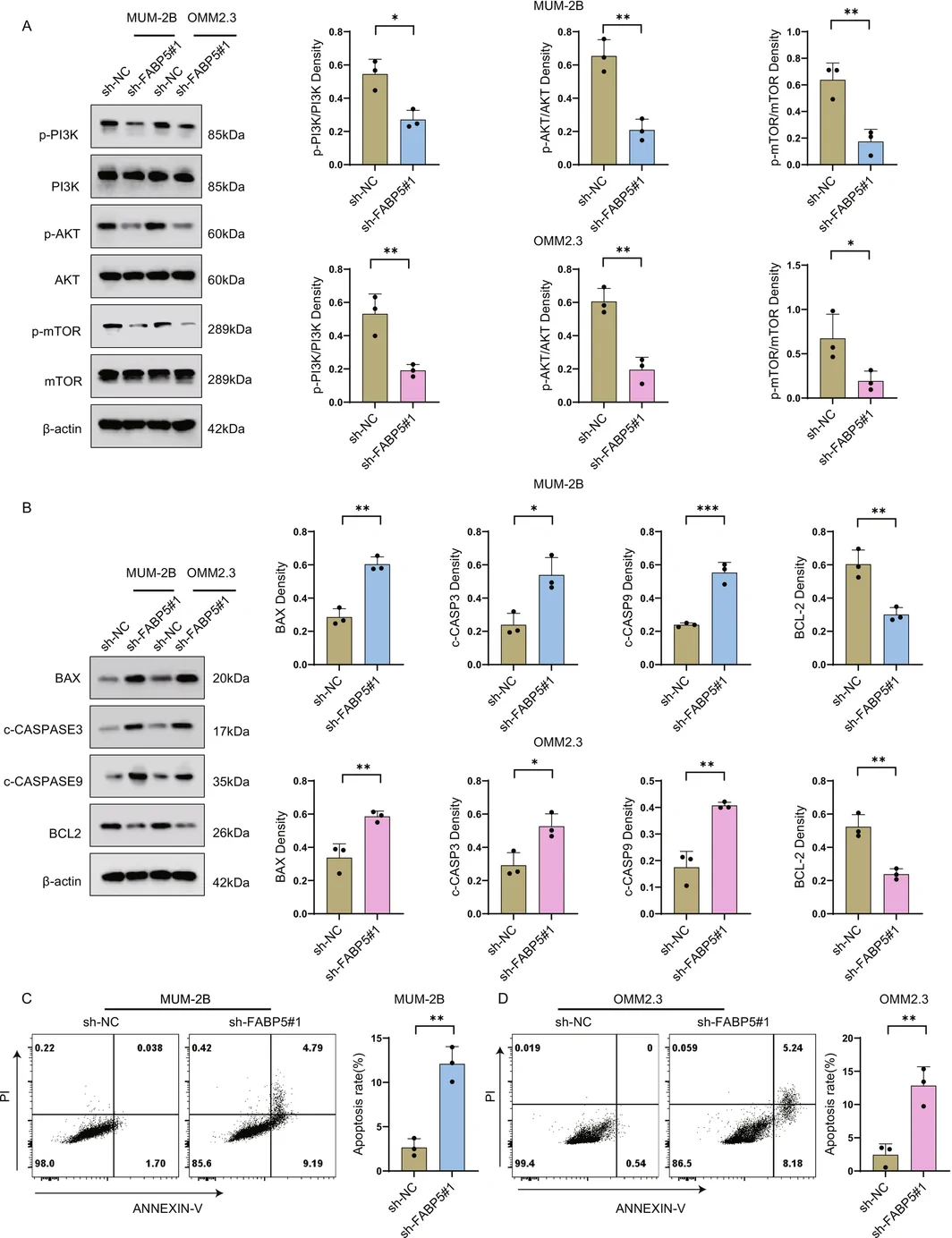
Protein expression profiles after sh‐*FABP5* transfection. (A) Expression levels of PI3K/AKT/mTOR pathway‐related molecules after sh‐*FABP5* transfection in MUM‐2B and OMM2.3 cell lines. (B) Expression levels of apoptosis‐related molecules after sh‐*FABP5* transfection in MUM‐2B and OMM2.3 cell lines. (C‐D) Analysis of apoptosis in (C) MUM‐2B and (D) OMM2.3 cells after sh‐*FABP5* transfection. * means *p* < 0.05, ** means *p* < 0.01, *** means *p* < 0.001.

In metastatic UVM cell lines, sh‐*FABP5*#1 transfection significantly reduced the levels of molecules related to the PI3K/AKT/mTOR pathway compared with the sh‐NC group (*p* < 0.05, Figure 3A), suggesting that *FABP5* may influence UVM progression via this signaling pathway. The sh‐*FABP5*#1 group also showed markedly higher expression of pro‐apoptosis‐related molecules than the sh‐NC group (*p* < 0.05, Figure 3B). Consistently, flow cytometry analysis revealed that apoptosis was notably higher in the sh‐*FABP5*#1 group in metastatic UVM cells compared with the sh‐NC group (*p* < 0.05, Figure 3C).

### 3.6 Cellular Changes After Treatment With PI3K Activator 740Y‐P

**FIGURE 4:**
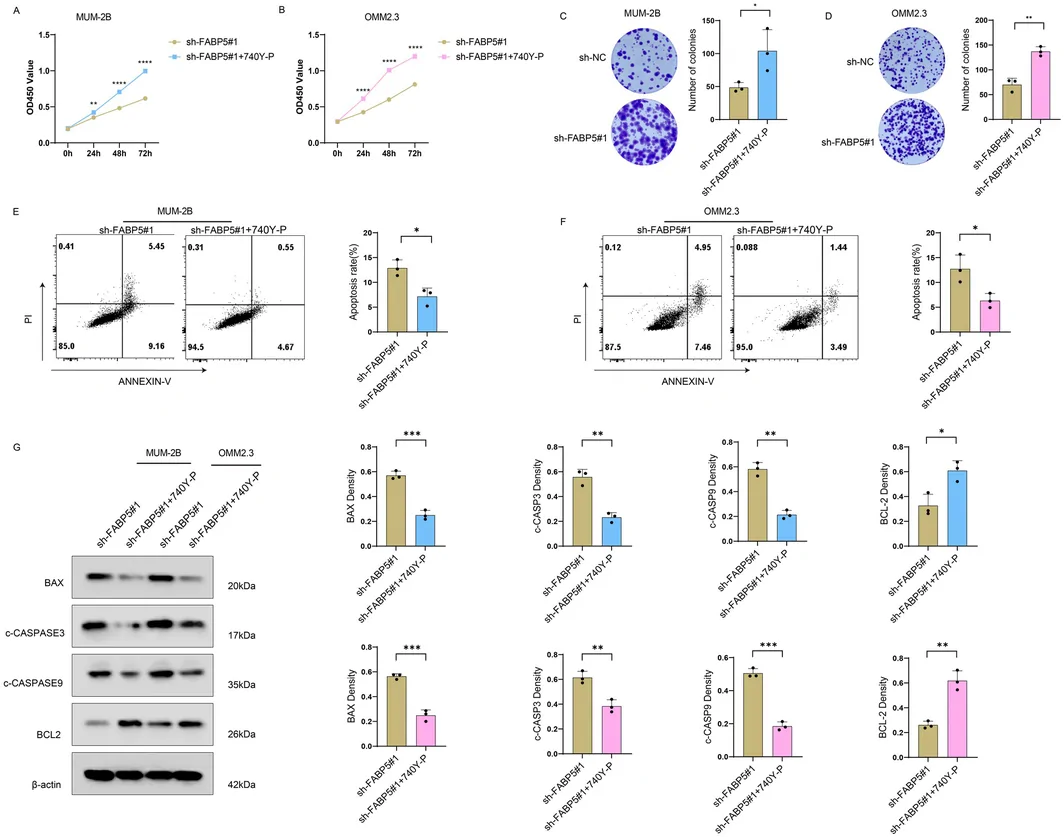
Cellular changes after sh‐*FABP5* transfection with 740Y‐P treatment. (A‐B) Cell viability after sh‐*FABP5* transfection plus 740Y‐P in (A) MUM‐2B and (B) OMM2.3 cell lines. (C‐D) Colony formation assay for proliferative capacity after sh‐*FABP5* transfection plus 740Y‐P in (C) MUM‐2B and (D) OMM2.3 cell lines. (E‐F) Flow cytometry analysis of apoptosis after sh‐*FABP5* transfection plus 740Y‐P in (E) MUM‐2B and (F) OMM2.3 cell lines. (G) Expression levels of apoptosis‐related molecules after sh‐*FABP5* transfection plus 740Y‐P in MUM‐2B and OMM2.3 cell lines. * means *p* < 0.05, ** means *p* < 0.01, *** means *p* < 0.001, **** means *p* < 0.0001.

CCK‐8 assays revealed that the viability of OMM2.3 and MUM‐2B co‐treated with sh‐*FABP5*#1 and 740Y‐P was markedly higher than that in the sh‐*FABP5*#1 group (*p* < 0.05, Figure 4A,B). Colony formation experiments demonstrated significantly increased proliferation in the sh‐*FABP5*#1 + 740Y‐P cells compared with sh‐*FABP5*#1 cells (*p* < 0.05, Figure 4C,D). Flow cytometry analysis showed that the apoptosis rate was significantly lower in the sh‐*FABP5*#1 + 740Y‐P groups than in the sh‐FABP5#1 group (*p* < 0.05, Figure 4E,F). The expression of pro‐apoptosis‐related molecules was markedly downregulated in the sh‐*FABP5*#1 + 740Y‐P group compared with the sh‐*FABP5*#1 group (*p* < 0.05, Figure 4G).

**FIGURE 5:**
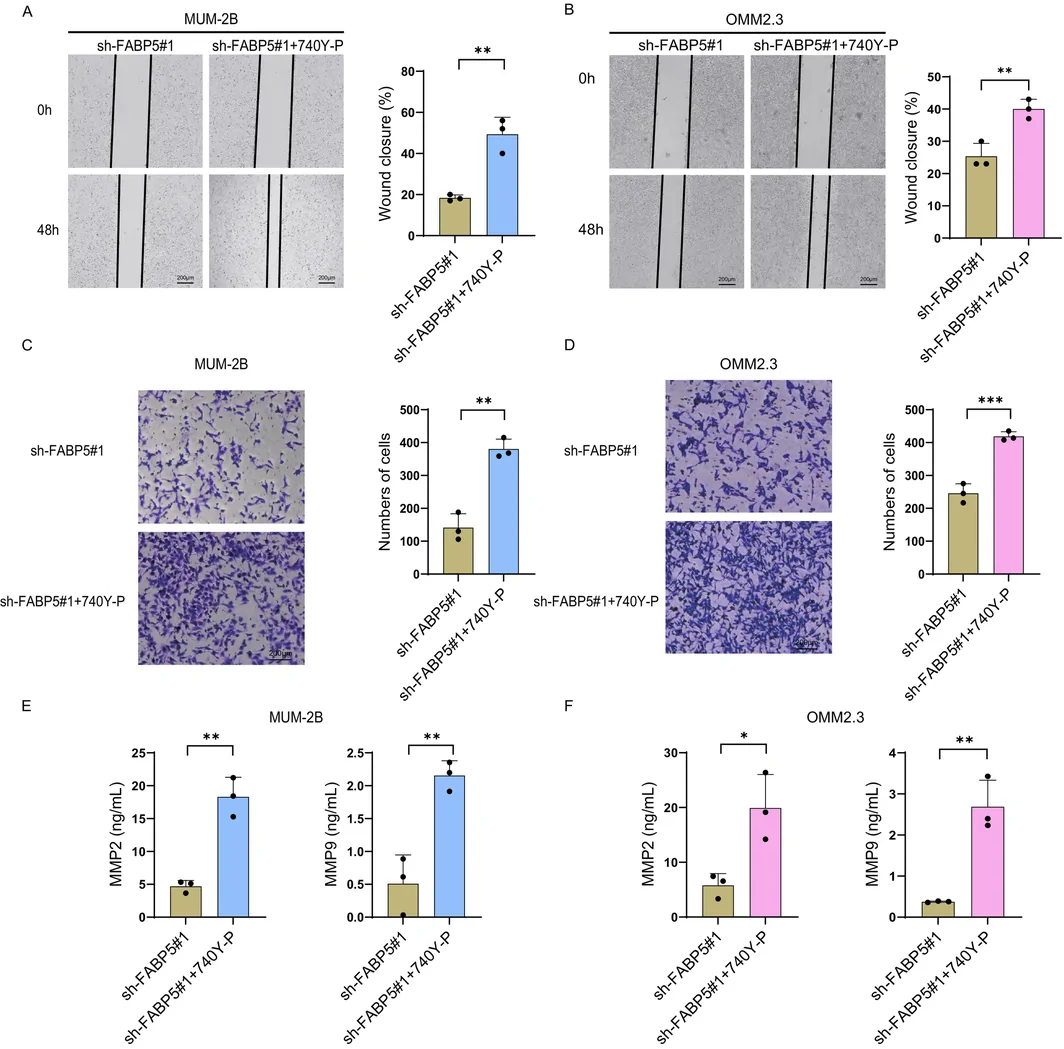
Cellular phenotypes after sh‐*FABP5* transfection plus 740Y‐P treatment. (A‐B) Wound healing assay for migration ability after sh‐*FABP5* transfection plus 740Y‐P in (A) MUM‐2B and (B) OMM2.3 cell lines. (C‐D) Transwell invasion assay for invasive capacity after sh‐*FABP5* transfection plus 740Y‐P in (C) MUM‐2B and (D) OMM2.3 cell lines. (E‐F) Level of MMP2 and MMP9 after sh‐*FABP5* transfection plus 740Y‐P in (E) MUM‐2B and (F) OMM2.3 cell lines. ** means *p* < 0.01, *** means *p* < 0.001.

Wound healing assay showed that the migration was significantly enhanced in the sh‐*FABP5*#1 + 740Y‐P group (*p* < 0.05, Figure 5A,B), and Transwell invasion assay confirmed a substantial increase in cell invasion (*p* < 0.05, Figure 5C,D). Additionally, *MMP2* and *MMP9* level was notably upregulated in the sh‐*FABP5*#1 + 740Y‐P group compared with the sh‐*FABP5*#1 group (*p* < 0.05, Figure 5E,F). These results indicated that PI3K activator 740Y‐P reversed the tumor‐suppressive effects of sh‐*FABP5* in UVM cells.

### 3.7 The Role of *FABP5* in the Mouse Tumor Models

**FIGURE 6:**
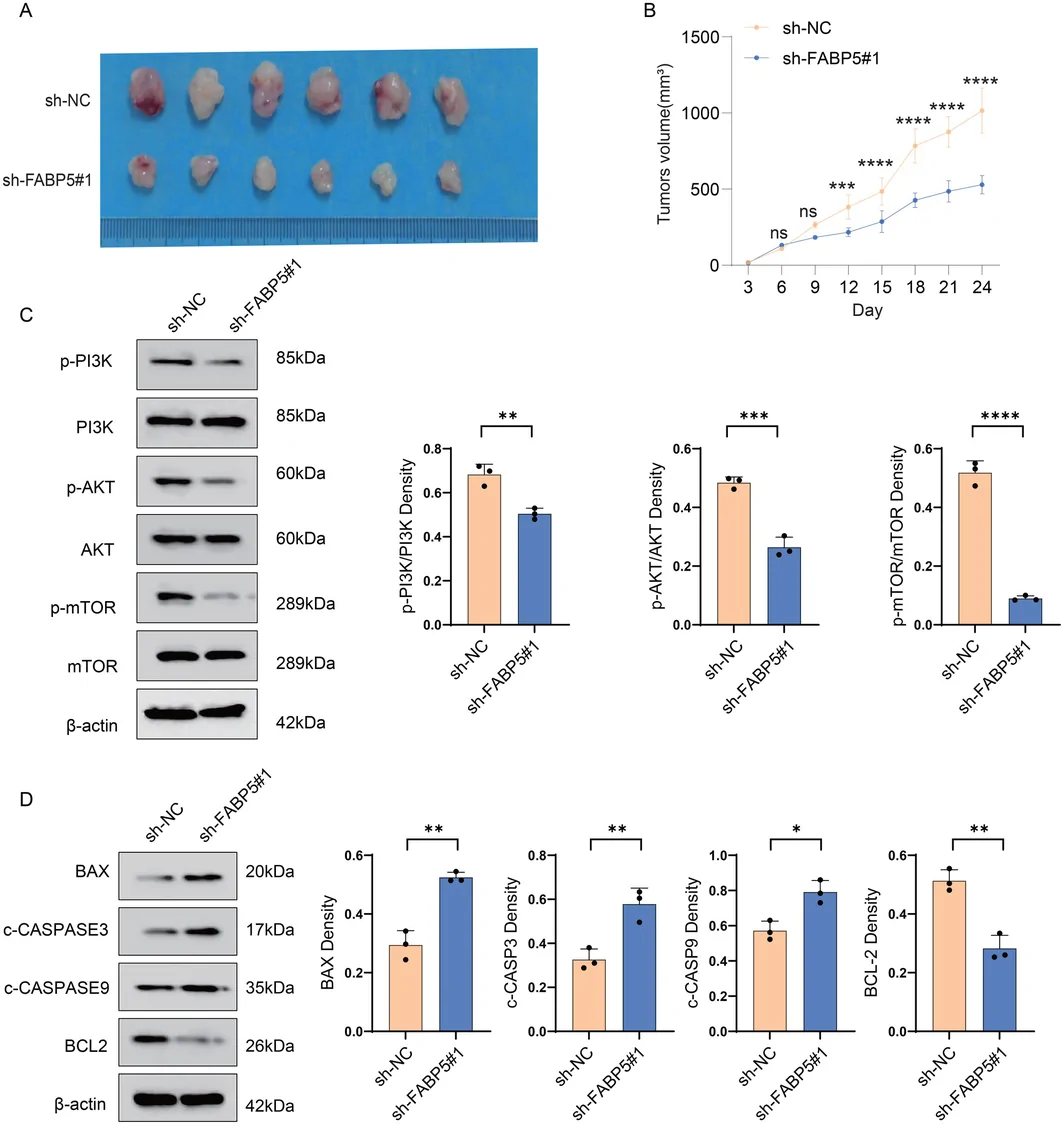
Establishment of mouse model and investigation of sh‐*FABP5*‐transfected tumor cells. (A) Tumor morphology in mice before and after sh‐*FABP5*#1 transfection. (B) Dynamic changes in tumor volume after sh‐*FABP5*#1 transfection. (C) Expression levels of PI3K/AKT/mTOR pathway‐related molecules in mouse tumors after sh‐*FABP5*#1 transfection. (D) Expression levels of apoptosis‐related molecules in mouse tumors after sh‐*FABP5*#1 transfection. ns means not significant, * means *p* < 0.05, ** means *p* < 0.01, *** means *p* < 0.001, **** means *p* < 0.0001.

In the xenograft tumor model, tumor volume in the sh‐*FABP5*#1 group was notably smaller than that in the sh‐NC group (*p* < 0.05, Figure 6A,B). WB analysis revealed that the levels of PI3K/AKT/mTOR pathway‐related molecules were markedly lower in the sh‐*FABP5*#1 group compared with the sh‐NC group (*p* < 0.05, Figure 6C). Consistently, the expression of pro‐apoptosis‐related molecules was significantly upregulated in the sh‐*FABP5*#1 group compared with the sh‐NC group (*p* < 0.05, Figure 6D).

## 4 Discussion

In this study, we developed a prognostic risk model for UVM using computational approaches and identified *FABP5* as a gene significantly associated with patient prognosis. Cellular experiments and in vivo mouse xenograft experiments demonstrated that *FABP5* may be associated with the progression of UVM through activating the PI3K/AKT/mTOR signaling pathway, although further validation is still required. These findings provide novel insights into UVM biology and may inform future efforts to identify therapeutic targets within the PI3K/AKT/mTOR pathway.

The risk model comprised three key genes (*SPON2*, *MATK*, and *FABP5*). Spondin 2 (*SPON2*) is a secreted extracellular matrix (ECM) protein belonging to the F‐spondin family. Studies have demonstrated that *SPON2* promotes the infiltration of M2‐polarized tumor‐associated macrophages (TAMs) [50], implicating it in UVM metastasis [51]. In contrast, the role of megakaryocyte‐associated tyrosine kinase (*MATK*) gene in tumors remains poorly understood, warranting further investigation. Fatty acid‐binding protein 5 (*FABP5*) is an intracellular carrier of fatty acids involved in lipid metabolism and cell proliferation [15]. It is overexpressed in many malignancies, where its level is associated with tumor growth and metastasis [52]. In UVM, high *FABP5* expression has been associated with poor prognosis, including shorter overall survival (OS) and PFI [26]. However, that study was correlative and did not explore *FABP5* function. In our study, *FABP5* was specifically overexpressed in metastatic UVM lines (OMM2.3 and MUM‐2B) compared with the primary cell line Mel270, linking *FABP5* to the metastatic phenotype rather than to UVM. Moreover, silencing *FABP5* provided direct functional evidence that it drove, rather than merely accompanied, the malignant behaviors of UVM cells. These findings collectively position *FABP5* as a candidate mechanistic driver in UVM pathogenesis.

Our computational analyses identified *FABP5* as a prognostic gene for UVM, with its expression correlated with the RiskScore. We then confirmed a higher expression of *FABP5* in the metastatic (OMM2.3 and MUM‐2B) than in the primary (Mel270) UVM cells, suggesting an association between *FABP5* and the metastatic phenotype of UVM. Silencing *FABP5* inhibited cell proliferation, migration, and invasion and the secretion of *MMP2/MMP9*, while promoting apoptosis. The observed reduction in *MMP2* and *MMP9* is consistent with earlier UVM studies linking these gelatinases to invasion and liver colonization [53], as well as with reports in other tumors implicating *FABP5* in MMP2‐dependent metastasis [21]. Collectively, these data position *FABP5* as a regulator of a coordinated program of proliferation, invasion, and cell survival in UVM.

We next examined the mechanism by which *FABP5* exerted these effects. Aberrant activation of the PI3K/AKT/mTOR pathway is closely linked to the development and progression of UVM [27]. This pathway not only drives tumor cell proliferation but also promotes chemoresistance and EMT‐mediated metastasis. Its importance in UVM has a clear molecular basis: most UVM cases carry activating *GNAQ* or *GNA11* mutations, and recent work has shown that Gαq signaling activates PI3K/AKT through focal adhesion kinase, with UVM cells depending on PI3K/AKT for survival [54]. In our study, silencing *FABP5* reduced PI3K/AKT/mTOR pathway‐related protein levels in both metastatic cell lines and xenograft tumors, increased the levels of pro‐apoptotic proteins, and downregulated the anti‐apoptotic protein Bcl‐2. This pattern aligns with reports in other cancers, where *FABP5* enhances PI3K/AKT/mTOR signaling [19]. Inhibiting *FABP5* reduces phosphorylation of PPARγ and AKT and increases cleaved caspase‐9 and caspase‐3 [55]. Blocking the PI3K/AKT/mTOR pathway also inhibits UVM migration, invasion, and survival [56]. To test whether *FABP5* acted through this pathway, we used the PI3K activator 740Y‐P following *FABP5* knockdown. Treatment with the PI3K agonist 740Y‐P rescued the proliferative, migratory, and invasive capacities as well as MMP expression in the sh‐*FABP5* cells and alleviated apoptosis. These results suggest that *FABP5* acts as the upstream of the PI3K/AKT/mTOR pathway and drives the malignant phenotype of UVM in a pathway‐dependent manner. As fatty acid‐binding proteins can be inhibited pharmacologically and such inhibition slows tumor growth in other cancers [57], pharmacological inhibition of PI3K may interfere with *FABP5* function to suppress UVM progression via blockade of the PI3K/AKT/mTOR signaling pathway, offering a potential therapeutic strategy. Nevertheless, this hypothesis requires verification in clinical trials.

This study found that the high‐risk group exhibited significant immune microenvironment remodeling, and that *FABP5* expression was closely related to the immune score. Previous studies in pancreatic neuroendocrine neoplasms have shown that targeted inhibition of *RTN3* upregulates *FABP5*, which in turn activates the PI3K/AKT/mTOR signaling pathway and promotes M2‐like macrophage polarization via fatty acid oxidation. These polarized M2 macrophages subsequently secrete *MMP2*, contributing to a metastasis‐promoting tumor microenvironment [21]. Additional evidence across other malignancies has confirmed a positive correlation between *FABP5* expression and macrophage infiltration, particularly M2‐type macrophages [58]. Collectively, these findings suggest that *FABP5* may modulate immune‐related biological processes through the PI3K/AKT/mTOR pathway, thereby contributing to tumor initiation and metastatic progression. Nevertheless, the present immune results were derived entirely from computational predictions; therefore, the hypothesis that *FABP5* or immune‐infiltration status could guide immunotherapy still requires experimental validation, particularly in preclinical models.

Several limitations in this research should be acknowledged. First, mechanistic validation was primarily based on cell lines and mouse models, lacking direct verification in large clinical cohorts, which may limit the generalizability of our conclusions. Future studies with larger sample sizes and more diverse UVM cohorts are needed to validate these findings. Second, the combination of *FABP5*‐targeted therapy with existing clinical regimens (e.g., ICIs) has not been explored, which limits the feasibility of clinical translation. Furthermore, the immunotherapy‐related findings were derived solely from computational predictions and have not been validated by any therapeutic agent. Future studies are encouraged to evaluate *FABP5*‐targeted approaches, both alone and in combination with immunotherapy.

## 5 Conclusion

In this study, a prognostic model for UVM comprising *SPON2*, *MATK*, and *FABP5* was constructed through computational analyzes. Cellular and animal experiments further validated that *FABP5* promoted malignant proliferation, migration, and invasion while suppressing apoptosis in UVM cells by activating the PI3K/AKT/mTOR pathway, effects that were reversed by the PI3K agonist 740Y‐P. Beyond clarifying the potential role of the FABP5/PI3K/AKT/mTOR signaling axis in UVM tumorigenesis and progression, these findings support the hypothesis that risk stratification based on immune infiltration may help guide immunotherapy selection in UVM, although this hypothesis remains to be confirmed in treated patient cohorts.

## Author Contributions

All authors contributed to this present work: [YD] and [QYY] designed the study, [YYZ] acquired the data, [JNY] interpreted the data. [XJ] drafted the manuscript, [YD] and [QYY] revised the manuscript. All authors read and approved the manuscript.

## Funding

The study was supported by Health Science and Technology Plan Project of Ningbo (2022Y42), Natural Science Foundation of Ningbo Municipality (2023J209), Medical Science and Technology Project of Zhejiang Province (2024KY367), and Medical Science and Technology Project of Zhejiang Province (2025KY1460).

## Ethics Statement

This study was approved by the Laboratory Animal Ethics Committee of Ningbo University (approval number: 14764).

## Consent

The authors have nothing to report.

## Conflicts of Interest

The authors declare no conflicts of interest.

## Supporting information


**Figure S1:** Characterization of immune microenviroment and prognostic value T cell subsets in UVM.
(A) The proportion of different cell types in metastatic and primary UVM. (B) the percentage of cell subsets between metastatic and primary UVM. (C) The Hazard Ration (HR) and *p* value for 3 types T cells regard prognosis. (D) Kaplan‐Meier survival curves analyzing the association between Cytotoxic T cell infiltration levels and Progression‐Free Interval (PFI) . (E) The enrichment scores of functional pathways across 3 types T cells.

**Figure S2:** Construction and validation of the risk model.
(A) The procedure for selecting the λ value in the LASSO regression model, illustrating how gene coefficients vary with the L1 norm. Identification of the optimal λ value through cross‐validation. (B) Three key genes and their coefficients. (C) A forest graph illustrating the hazard ratios of these genes along with their 95% confidence intervals. (D‐F) (D) 1–5 year‐ROC curves, (E) PFI differences between two risk groups, and (F) expression of key genes in the TCGA cohort. (G‐I) (G) 1–5 year‐ROC curves, (H) PFI differences between two risk groups, and (I) expression of key genes in GSE22138 cohort. * means *p* < 0.05, *** means *p* < 0.001, **** means *p* < 0.0001.

**Figure S3:** Response to immunotherapy in different risk groups.
(A) Differences in ESTIMATE scores between risk subgroups. (B) Correlation between three key genes and ESTIMATE immune scores in the TCGA cohort. (C) The ssGSEA score differences of 28 immune cell types between risk groups in the TCGA cohort. (D) Correlation between RiskScore and TIDE scores in the TCGA cohort. (E) CIBERSORT score differences between risk groups in the TCGA cohort. (F) Immunotherapeutic response to ICI between risk subgroups. (G) Comparison of TIDE scores between risk subgroups. (H) Distribution differences in TMB between risk subgroups. (I) Heatmap of immune checkpoint expression between risk subgroups. ns means not significant, * means *p* < 0.05, ** means *p* < 0.01, *** means *p* < 0.001.

**Figure S4:** GSEA.
(A) GSEA RESULTS BETWEEN RISK SUBGROUPS. (B) CORRELATION ANALYSIS BETWEEN THREE KEY GENES AND HALLMARK PATHWAY.

**Table S1:** THE PRIMARY ANTIBODY UTILIZED IN THIS STUDY.

## Data Availability

The datasets generated and/or analyzed during the current study are available in the [GSE22138] repository, [https://www.ncbi.nlm.nih.gov/geo/query/acc.cgi?acc=GSE22138] and [GSE139829] repository, [https://www.ncbi.nlm.nih.gov/geo/query/acc.cgi?acc=GSE139829].
