# Supplementary material for: Fatty Acid‐Binding Protein 5 Potentially Regulates the Proliferation and Metastasis of Uveal Melanoma Cells Through the PI3K/AKT/mTOR Signaling Pathway
Source: CNS Neurosci Ther. 2026 Sep 4;32(9):e71112. doi: 10.1002/cns.71112 (PMC13542988; doi:10.1002/cns.71112)
Supplement: Supplementary file 1 [file CNS-32-e71112-s001.pdf]

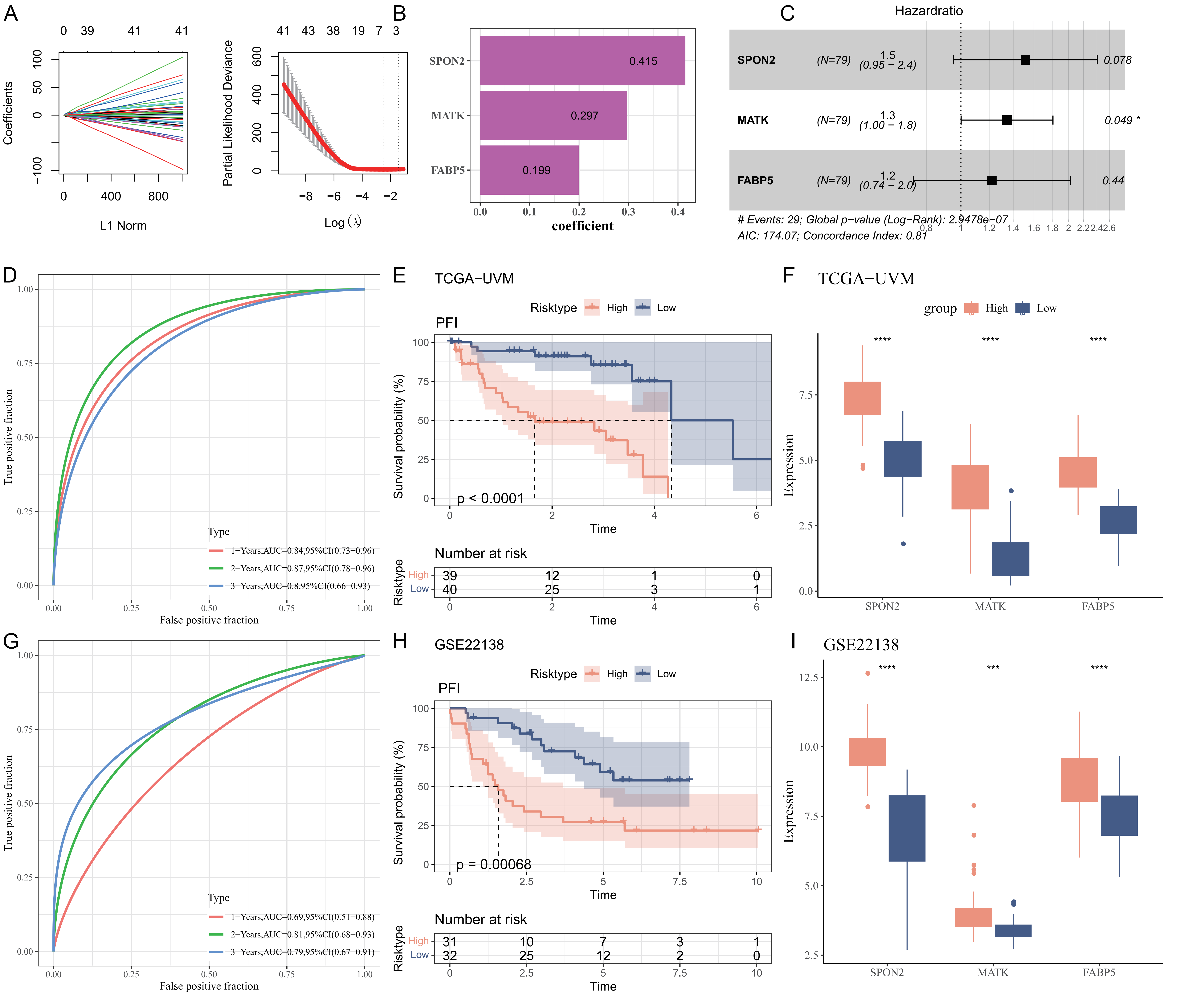

A
B
C
Hazardratio
SPON2 (N=79) 1.5 (0.95 − 2.4) 0.078
MATK (N=79) 1.3 (1.00 − 1.8) 0.049 *
FABP5 (N=79) 1.2 (0.74 − 2.0) 0.44
Events: 29; Global p−value (Log−Rank): 2.9478e−07
AIC: 174.07; Concordance Index: 0.81
L1 Norm
Coefficients
Partial Likelihood Deviance
Log(λ)
coefficient
0.415
0.297
0.199
D
E
F
TCGA−UVM
True positive fraction
False positive fraction
Type
1−Years,AUC=0.84,95%CI(0.73−0.96)
2−Years,AUC=0.87,95%CI(0.78−0.96)
3−Years,AUC=0.8,95%CI(0.66−0.93)
Risktype High Low
PFI
Survival probability (%)
p < 0.0001
Time
Number at risk
High 39 12 1 0
Low 40 25 3 1
group High Low
Expression
SPON2 MATK FABP5
G
H
I
GSE22138
1−Years,AUC=0.69,95%CI(0.51−0.88)
2−Years,AUC=0.81,95%CI(0.68−0.93)
3−Years,AUC=0.79,95%CI(0.67−0.91)
p = 0.00068
High 31 10 7 3 1
Low 32 25 12 2 0
