# Supplementary material for: Fatty Acid‐Binding Protein 5 Potentially Regulates the Proliferation and Metastasis of Uveal Melanoma Cells Through the PI3K/AKT/mTOR Signaling Pathway
Source: CNS Neurosci Ther. 2026 Sep 4;32(9):e71112. doi: 10.1002/cns.71112 (PMC13542988; doi:10.1002/cns.71112)
Supplement: Supplementary file 2 [file CNS-32-e71112-s002.pdf]

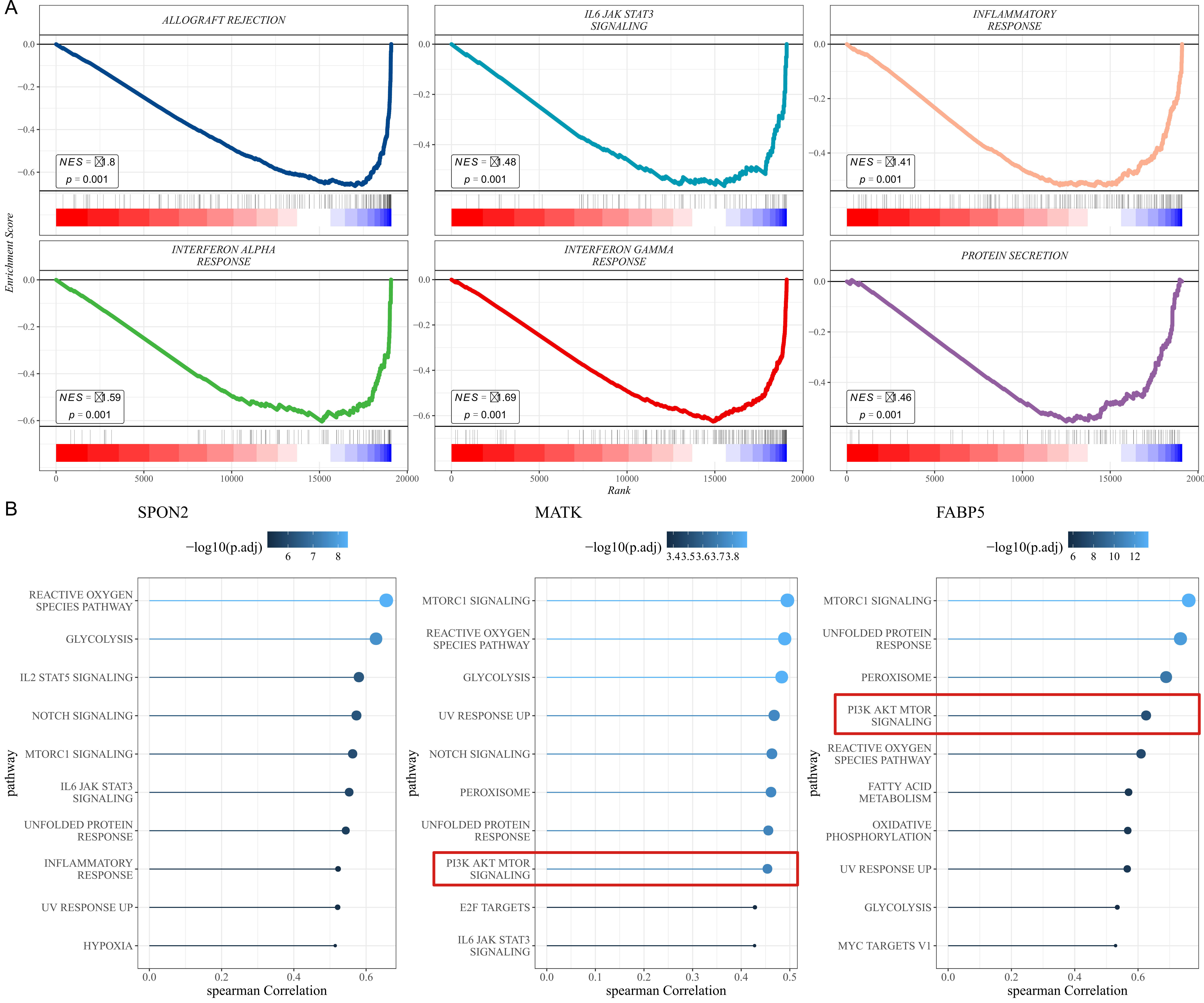

A
ALLOGRAFT REJECTION
NES = ⊠1.8
p = 0.001
IL6 JAK STAT3 SIGNALING
NES = ⊠1.48
p = 0.001
INFLAMMATORY RESPONSE
NES = ⊠1.41
p = 0.001
INTERFERON ALPHA RESPONSE
NES = ⊠1.59
p = 0.001
INTERFERON GAMMA RESPONSE
NES = ⊠1.69
p = 0.001
PROTEIN SECRETION
NES = ⊠1.46
p = 0.001
Enrichment Score
Rank
B
SPON2
−log10(p.adj)
REACTIVE OXYGEN SPECIES PATHWAY
GLYCOLYSIS
IL2 STAT5 SIGNALING
NOTCH SIGNALING
MTORC1 SIGNALING
IL6 JAK STAT3 SIGNALING
UNFOLDED PROTEIN RESPONSE
INFLAMMATORY RESPONSE
UV RESPONSE UP
HYPOXIA
pathway
spearman Correlation
MATK
−log10(p.adj)
MTORC1 SIGNALING
REACTIVE OXYGEN SPECIES PATHWAY
GLYCOLYSIS
UV RESPONSE UP
NOTCH SIGNALING
PEROXISOME
UNFOLDED PROTEIN RESPONSE
PI3K AKT MTOR SIGNALING
E2F TARGETS
IL6 JAK STAT3 SIGNALING
pathway
spearman Correlation
FABP5
−log10(p.adj)
MTORC1 SIGNALING
UNFOLDED PROTEIN RESPONSE
PEROXISOME
PI3K AKT MTOR SIGNALING
REACTIVE OXYGEN SPECIES PATHWAY
FATTY ACID METABOLISM
OXIDATIVE PHOSPHORYLATION
UV RESPONSE UP
GLYCOLYSIS
MYC TARGETS V1
pathway
spearman Correlation
