# Supplementary material for: Fatty Acid‐Binding Protein 5 Potentially Regulates the Proliferation and Metastasis of Uveal Melanoma Cells Through the PI3K/AKT/mTOR Signaling Pathway
Source: CNS Neurosci Ther. 2026 Sep 4;32(9):e71112. doi: 10.1002/cns.71112 (PMC13542988; doi:10.1002/cns.71112)
Supplement: Supplementary file 3 [file CNS-32-e71112-s003.pdf]

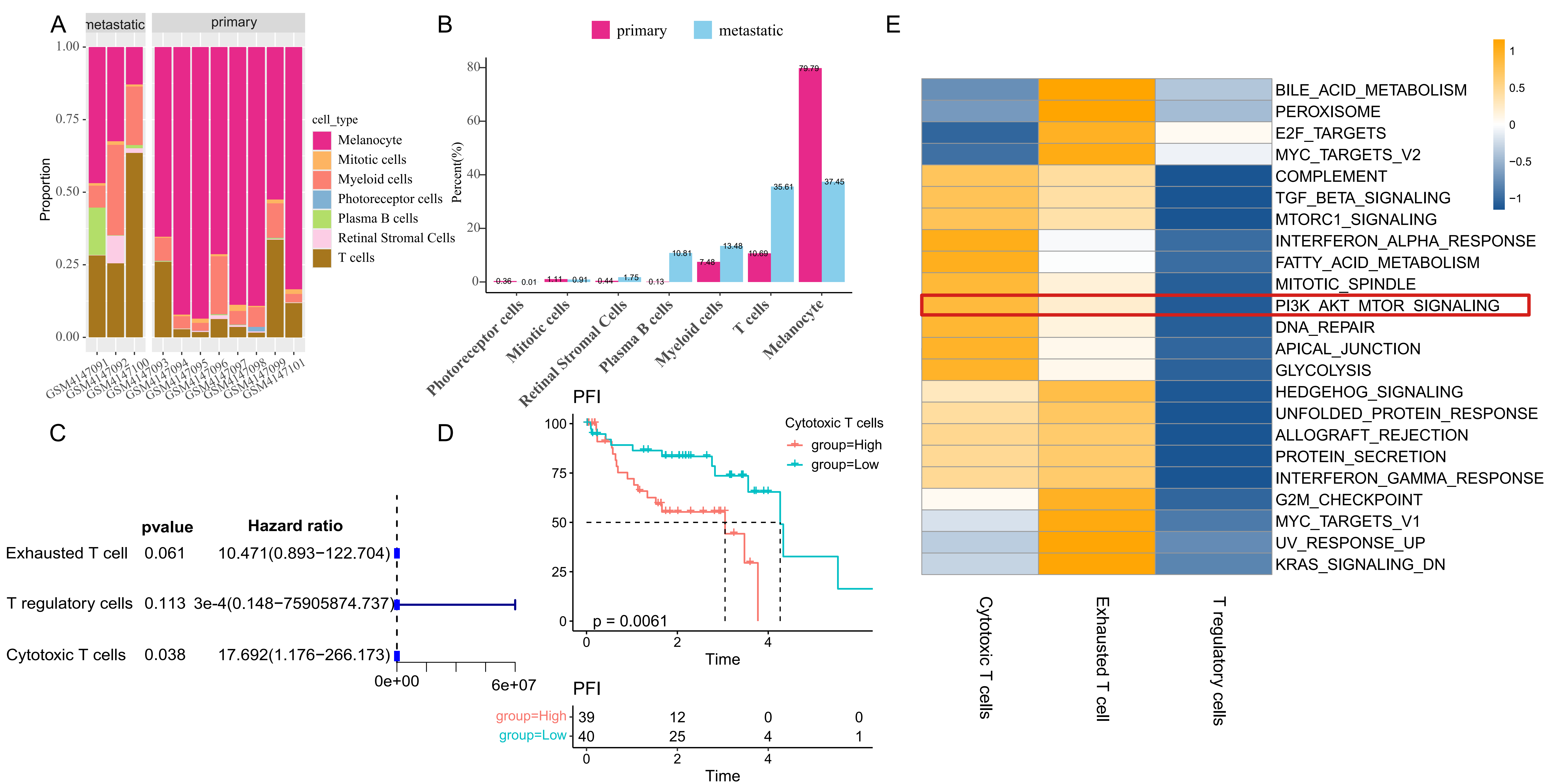
A
metastatic
primary
Proportion
1.00
0.75
0.50
0.25
0.00
GSM4147091
GSM4147092
GSM4147100
GSM4147093
GSM4147094
GSM4147095
GSM4147096
GSM4147097
GSM4147098
GSM4147099
GSM4147101
cell_type
Melanocyte
Mitotic cells
Myeloid cells
Photoreceptor cells
Plasma B cells
Retinal Stromal Cells
T cells
B
primary
metastatic
Percent(%)
80
60
40
20
0
0.36
0.01
1.11
0.91
0.44
1.75
0.13
10.81
7.48
13.48
10.69
35.61
79.79
37.45
Photoreceptor cells
Mitotic cells
Retinal Stromal Cells
Plasma B cells
Myeloid cells
T cells
Melanocyte
C
pvalue
Hazard ratio
Exhausted T cell
0.061
10.471(0.893−122.704)
T regulatory cells
0.113
3e-4(0.148−75905874.737)
Cytotoxic T cells
0.038
17.692(1.176−266.173)
0e+00
6e+07
D
PFI
Cytotoxic T cells
group=High
group=Low
100
75
50
25
0
p = 0.0061
0
2
4
Time
PFI
group=High 39 12 0 0
group=Low 40 25 4 1
0
2
4
Time
E
BILE_ACID_METABOLISM
PEROXISOME
E2F_TARGETS
MYC_TARGETS_V2
COMPLEMENT
TGF_BETA_SIGNALING
MTORC1_SIGNALING
INTERFERON_ALPHA_RESPONSE
FATTY_ACID_METABOLISM
MITOTIC_SPINDLE
PI3K_AKT_MTOR_SIGNALING
DNA_REPAIR
APICAL_JUNCTION
GLYCOLYSIS
HEDGEHOG_SIGNALING
UNFOLDED_PROTEIN_RESPONSE
ALLOGRAFT_REJECTION
PROTEIN_SECRETION
INTERFERON_GAMMA_RESPONSE
G2M_CHECKPOINT
MYC_TARGETS_V1
UV_RESPONSE_UP
KRAS_SIGNALING_DN
1
0.5
0
−0.5
−1
Cytotoxic T cells
Exhausted T cell
T regulatory cells
