# Supplementary material for: Fatty Acid‐Binding Protein 5 Potentially Regulates the Proliferation and Metastasis of Uveal Melanoma Cells Through the PI3K/AKT/mTOR Signaling Pathway
Source: CNS Neurosci Ther. 2026 Sep 4;32(9):e71112. doi: 10.1002/cns.71112 (PMC13542988; doi:10.1002/cns.71112)
Supplement: Supplementary file 4 [file CNS-32-e71112-s005.pdf]

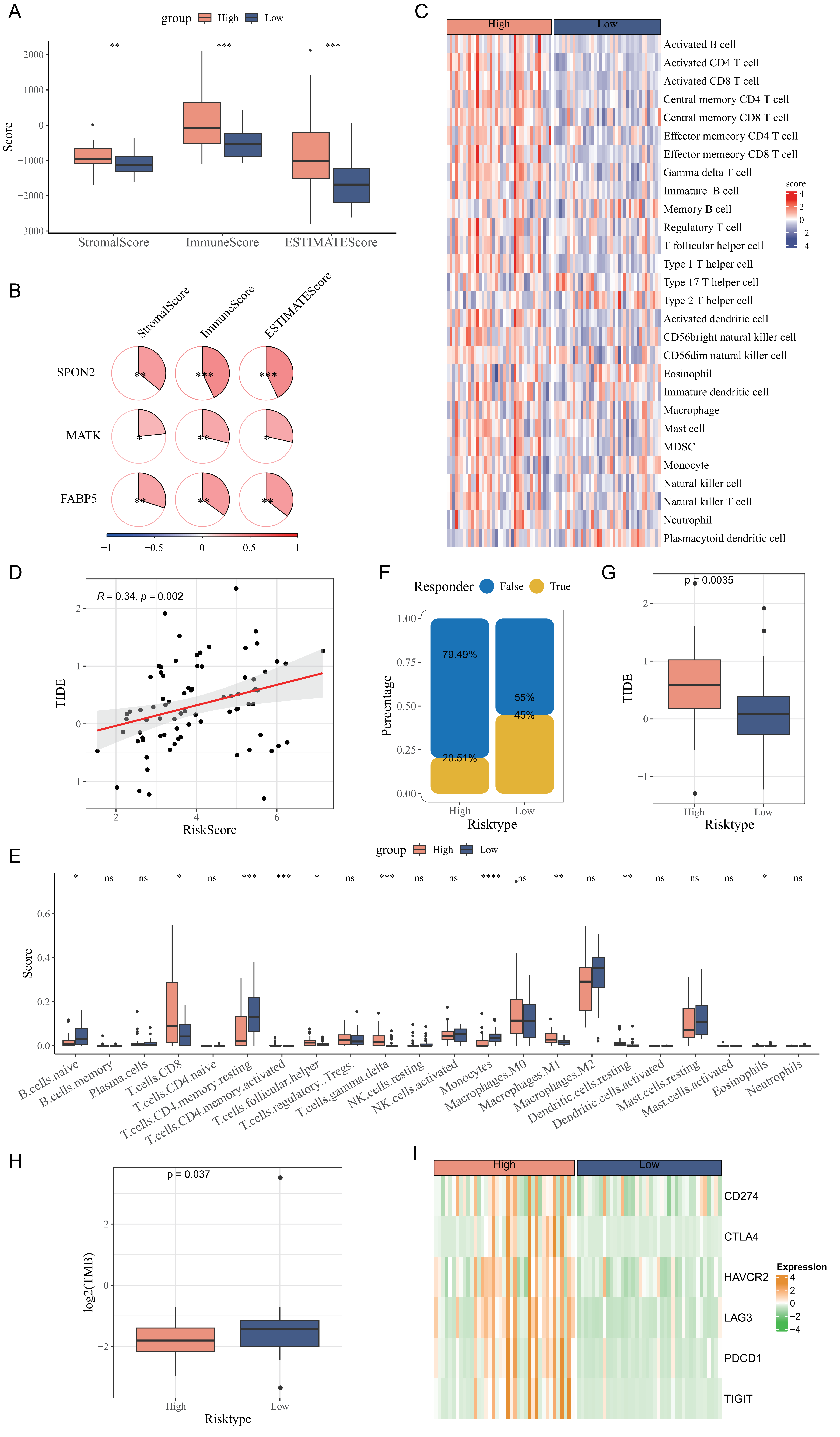

A
group High Low
** *** ***
Score
StromalScore ImmuneScore ESTIMATEScore
B
StromalScore ImmuneScore ESTIMATEScore
SPON2
MATK
FABP5
C
High Low
Activated B cell
Activated CD4 T cell
Activated CD8 T cell
Central memory CD4 T cell
Central memory CD8 T cell
Effector memeory CD4 T cell
Effector memeory CD8 T cell
Gamma delta T cell
Immature B cell
Memory B cell
Regulatory T cell
T follicular helper cell
Type 1 T helper cell
Type 17 T helper cell
Type 2 T helper cell
Activated dendritic cell
CD56bright natural killer cell
CD56dim natural killer cell
Eosinophil
Immature dendritic cell
Macrophage
Mast cell
MDSC
Monocyte
Natural killer cell
Natural killer T cell
Neutrophil
Plasmacytoid dendritic cell
score
D
R = 0.34, p = 0.002
TIDE
RiskScore
F
Responder False True
79.49%
20.51%
55%
45%
Percentage
High Low
Risktype
G
p = 0.0035
TIDE
High Low
Risktype
E
group High Low
Score
B.cells.naive
B.cells.memory
Plasma.cells
T.cells.CD8
T.cells.CD4.naive
T.cells.CD4.memory.resting
T.cells.CD4.memory.activated
T.cells.follicular.helper
T.cells.regulatory..Tregs.
T.cells.gamma.delta
NK.cells.resting
NK.cells.activated
Monocytes
Macrophages.M0
Macrophages.M1
Macrophages.M2
Dendritic.cells.resting
Dendritic.cells.activated
Mast.cells.resting
Mast.cells.activated
Eosinophils
Neutrophils
H
p = 0.037
log2(TMB)
High Low
Risktype
I
High Low
CD274
CTLA4
HAVCR2
LAG3
PDCD1
TIGIT
Expression
