# Supplementary material for: Fatty Acid‐Binding Protein 5 Potentially Regulates the Proliferation and Metastasis of Uveal Melanoma Cells Through the PI3K/AKT/mTOR Signaling Pathway
Source: CNS Neurosci Ther. 2026 Sep 4;32(9):e71112. doi: 10.1002/cns.71112 (PMC13542988; doi:10.1002/cns.71112)
Supplement: Supplementary file 5 [file CNS-32-e71112-s004.docx]

Table S1. The primary antibody utilized in this study.

| Gene | Cat. | Company | Dilution ratio |
| --- | --- | --- | --- |
| FABP5 | ab255276 | Abcam | 1:1000 |
| GAPDH | ab9485 | Abcam | 1:2500 |
| p-PI3K | ab138364 | Abcam | 1:1000 |
| PI3K | 4292 | Cell Signaling Technology | 1:1000 |
| p-AKT | 4060 | Cell Signaling Technology | 1:2000 |
| AKT | ab8805 | Abcam | 1:1000 |
| p-mTOR | ab109268 | Abcam | 1:10000 |
| mTOR | ab134903 | Abcam | 1:10000 |
| Bax | 2772 | Cell Signaling Technology | 1:5000 |
| cleaved caspase-3 | 9661 | Cell Signaling Technology | 1:1000 |
| cleaved caspase-9 | 9505 | Cell Signaling Technology | 1:1000 |
| Bcl-2 | 4223 | Cell Signaling Technology | 1::1000 |
| β-actin | ab8227 | Abcam | 1:2000 |
